# A Preliminary Study for Evaluating the Dose-Dependent Effect of d-Allulose for Fat Mass Reduction in Adult Humans: A Randomized, Double-Blind, Placebo-Controlled Trial

**DOI:** 10.3390/nu10020160

**Published:** 2018-01-31

**Authors:** Youngji Han, Eun-Young Kwon, Mi Kyeong Yu, Seon Jeong Lee, Hye-Jin Kim, Seong-Bo Kim, Yang Hee Kim, Myung-Sook Choi

**Affiliations:** 1 Department of Food Science and Nutrition, Kyungpook National University, 80 Daehakro, Bukgu, Daegu 41566, Korea; youngji.kor.han@gmail.com (Y.H.); savagegarden01@hanmail.net (E.-Y.K.); yumeijing@naver.com (M.K.Y.); end0901@hanmail.net (S.J.L.); 2 Center for Food and Nutritional Genomics Research, Kyungpook National University, Daegu 41566, Korea; 3 Food R&D, CJ Cheiljedang Corp., 55, Gwanggyo-ro 42beon-gil, Yeongtong-gu, Suwon-si, Gyeonggi-do 16495, Korea; hyejin.kim@cj.net (H.-J.K.); seongbo.kim@cj.net (S.-B.K.); yanghee.kim@cj.net (Y.H.K.)

**Keywords:** d-allulose, sugar substitutes, obesity, randomized-controlled trial

## Abstract

d-allulose is a rare sugar with zero energy that can be consumed by obese/overweight individuals. Many studies have suggested that zero-calorie d-allulose has beneficial effects on obesity-related metabolism in mouse models, but only a few studies have been performed on human subjects. Therefore, we performed a preliminary study with 121 Korean subjects (aged 20–40 years, body mass index ≥ 23 kg/m^2^). A randomized controlled trial involving placebo control (sucralose, 0.012 g × 2 times/day), low d-allulose (d-allulose, 4 g × 2 times/day), and high d-allulose (d-allulose, 7 g × 2 times/day) groups was designed. Parameters for body composition, nutrient intake, computed tomography (CT) scan, and plasma lipid profiles were assessed. Body fat percentage and body fat mass were significantly decreased following d-allulose supplementation. The high d-allulose group revealed a significant decrease in not only body mass index (BMI), but also total abdominal and subcutaneous fat areas measured by CT scans compared to the placebo group. There were no significant differences in nutrient intake, plasma lipid profiles, markers of liver and kidney function, and major inflammation markers among groups. These results provide useful information on the dose-dependent effect of d-allulose for overweight/obese adult humans. Based on these results, the efficacy of d-allulose for body fat reduction needs to be validated using dual energy X-ray absorption.

## 1. Introduction

Obesity, a hallmark of the metabolic syndrome, has increased to epidemic proportions worldwide and become a leading cause of morbidity [1,2,3]. Obesity changes the general metabolism of the body, as well as its appearance [4]. It induces metabolic disorders such as type 2 diabetes, dyslipidemia, and cardiovascular diseases with inflammation [5]. According to the Korea National Health and Nutrition Examination Survey [6], the incidence of obesity (body mass index (BMI) ≥ 25 kg/m^2^) among adults over 19 years of age in Korea was 26% in 1998. Since then, it has been constantly increasing and reached 30.9% in 2014 (males, 37.7%; females, 23.3%). Further, the risk of developing hypertension, diabetes, and dyslipidemia was about twice as high in obese people as that in people with normal weight.

Many studies have suggested that sugar is one of the dietary factors that is responsible for obesity in the modern society [7,8,9]. Sugar substitutes have been receiving more attention than ever before [10,11,12]. Thus, it is essential to develop economical and safe sweeteners to replace sugar. d-allulose, C-3 epimer of d-fructose, is a sugar substitute, which has 70% of the sweetness of sucrose but almost zero calories and is rarely found in nature. It is only present in small quantities in commercial mixtures of d-glucose and d-fructose obtained from the hydrolysis of sucrose or isomerization of d-glucose [13]. However, currently, it can be manufactured in large quantities from the hydrolysis of sucrose or isomerization of d-glucose using enzymatic methods [14]. d-allulose is generally recognized as safe (GRAS), according to the United States department of agriculture (USDA) regulations [15]. Several studies have provided preliminary evidence on the impact of d-allulose on lipid metabolism [16,17,18,19]. Although d-allulose was suggested to act in a similar manner as d-fructose, interestingly, it enhances glucose uptake from liver and suppresses hepatic lipogenic enzyme activities [16]. In addition, it lowered food intake while it increased energy expenditure during darkness and soleus muscle lipoprotein lipase activity in rats pair-fed the high-sucrose diet [17]. d-allulose inhibits dietary fat absorption in the small intestine and increases β-oxidation in fat tissue under pair-feeding conditions in mice fed with a high-fat diet [18]. Most studies indicated that d-allulose induces a decrease in body weight, fat mass, and food or energy intake in experimental animal models [19]. However, there have been only a few studies that have been conducted in human subjects to test the efficacy of d-allulose, and they include some limitations in the form of small sizes of subjects and in the use of placebo material [20]. As such, we conducted a preliminary study with overweight and obese subjects with compensation. This preliminary study will provide information on the approximate daily dosage of d-allulose for the upcoming main study, which would be performed using dual energy X-ray absorptiometry (DEXA) equipment for body fat measurement.

## 2. Materials and Methods

Test materials were provided in a bottle, in the form of a grapefruit flavored non-carbonated drink (30 mL), containing either sucralose or d-allulose (Table 1). Participants consumed the placebo or d-allulose drinks (two bottles per day). In order to choose the doses of d-allulose used in our study, we translated the dose in our previous animal study on the basis of Reagan-Shaw et al.’s report in 2008 [18,20]. All drinks were supplied by CJ Cheiljedang, Suwon, Kyoung gi-do, Korea. Test drinks were identified by different code numbers, the identities of which were not revealed to the investigator or subjects until the completion of the study.

### 2.1. Subjects

Participants were recruited via an advertisement which was posted on the bulletin board in Kyungpook National University and online. All participants were aged 20–40 years old and resided in the Daegu city and its suburb areas in the Republic of Korea in March 2016. After an initial screening, 144 subjects with BMI ≥ 23 kg/m^2^ were selected. The exclusion criteria were as follows: (1) Hypertensive taking diuretics; (2) Taking oral hypoglycemic agents or insulin injection; (3) Serious cardiac, renal, hepatic, thyroid, or cerebrovascular disease; (4) Serious cystic or gastrointestinal disease, gout, or porphyria; (5) Psychiatric problems such as depressive disorder, schizophrenia, alcoholic, and drug intoxication; (6) Taking functional food products that may affect the results of this study; (7) A history of surgery within the past 6 months; (8) Cancer diagnosis and treatment; (9) Asthma or other allergies; (10) Pregnant or in lactation period. The study was approved by the Clinical Research Information System (CRIS) of the Ministry of Health and Welfare of Republic of Korea (CRiS, KCT0002084) and the Kyungpook National University Human Research Committee (KNU 2016-17). All subjects gave their written informed consent for inclusion before they participated in the study.

### 2.2. Sample Size

Sample size was estimated using G*Power 3.1.9.2. Assuming a statistical power of 95%, significance level of 0.05, and effected difference of 0.40 (Cohen’s standard large), it was estimated that at least 102 participants would be needed to show a statistically significant difference in biomarkers of body fat among three groups [21].

### 2.3. Design

This research was conducted from March to August 2016 as a randomized, double-blinded, parallel, placebo-controlled study. The random allocation sequence was created using computer generated random numbers. For randomization, all subjects were randomly assigned in a 1:1:1 ratio to the following three nutritional intervention groups: placebo control (*n* = 48), low d-allulose (*n* = 48), and high d-allulose (*n* = 48) groups. The mechanism used for allocation concealment was sequentially numbered containers by an independent laboratory researcher, and participants were kept blinded to the sequence and randomization until the end of the study. The supplement drinks containing d-allulose and placebo (sucralose) did not differ in flavor and color, and each participant received the supplement drinks in white plastic bottles. During the study, the participants were instructed to maintain their routine food intake and physical activity, and consume two bottles of supplements per day, with each bottle consumed after a meal, for 12 weeks (April to June 2016). At the end of study, subjects were asked to return any beverage bottles not consumed. The doses of d-allulose were selected by extrapolated calculations based on a previous animal study [18]. The primary outcome markers were body fat mass and body fat percentage, and secondary outcome markers were other body composition factors and plasma obesity-related biomarkers.

### 2.4. Anthropometric and Biochemical Analyses

For anthropometric and physiological measurements at baseline and 4, 8, and 12 weeks post-test material supplementation, subjects visited the Science Research Center Laboratory at Kyungpook National University between 07:00 and 11:00 h after a 12-h overnight fast. The waist circumference, hip circumference, blood pressure (BP), fasting blood glucose (FBG), glycosylated hemoglobin (HbA1c), and lipids were determined at baseline and after 12 weeks in the preliminary trial. The body mass index (BMI), height, weight, and body composition were measured using the X-Scan Plus II body composition analyzer (Jawon Medical Company, Daejeon, Korea). Brivo CT 385 (GE Healthcare, Chicago, IL, USA) was used for the computed assisted tomography (CT) scan, and the CT scans was taken at Doctors Radiology Clinic located in Daegu city. Among all participants, CT scans were only performed for subjects with agreement of radiation exposure. The waist and hip circumferences were measured with an anthropometric tape. The waist circumference was measured as the minimum circumference between the iliac crest and rib cage, and the hip circumference was measured as the maximum width over the greater trochanters. The waist-to-hip ratio (WHR) was calculated by dividing the waist measurement by the hip measurement. FBG, HbA1c, and blood pressure were measured using a glucose analyzer (LifeScan Inc., Milpitas, CA, USA), HbA1c analyzer (Micromat™ Hemoglobin A1c Test; Bio-Rad, Hercules, CA, USA), and automatic BP monitor (Omron, Kyoto, Japan), respectively. In addition, blood samples were collected in ethylenediaminetetraacetic acid (EDTA)-coated tubes and centrifuged at 1000× *g* for 15 min at 4 °C for plasma assays. Dietary intake was recorded using 24-h dietary recalls for each subject before and during the preliminary trial. Three-day dietary recalls were performed twice at baseline and follow up period, with a total of six 24-h dietary recalls by dietitians. We presented the mean of the three-day dietary intake in each point. The three-day dietary recalls were performed on non-consecutive three days which included two weekdays and one weekend day. Subjects were interviewed and asked what kinds of food they ate and drank on their dietary recall sheet. Nutrition food replicas were provided to help subjects estimate their dietary intakes with exact portions. Nutritional analysis was performed using the CAN-Pro 3.0 software (The Korean Nutrition Society, Seoul, Korea), which provides a comprehensive database for the nutritional content of general foods and special Korean foods. Physical activity levels were scored by the activity evaluation (1, Low; 2, Moderate; 3, High) which was calculated with multiplication of intensity, duration, and frequency.

### 2.5. Plasma Lipid Analyses

Plasma lipid concentrations were determined using commercially available kits for total cholesterol, triglycerides, high-density lipoprotein (HDL) cholesterol (Asan Pharm. Co., Seoul, Korea), and free fatty acids (FFA) (Wako Chemicals, Richmond, VA, USA). Low-density lipoprotein (LDL) cholesterol level was calculated using the Friedewald formula (total cholesterol—HDL cholesterol—(triglycerides/5)) [22]. Non-HDL cholesterol level was calculated as follows: HDL cholesterol—total cholesterol. The atherogenic index (AI) was calculated as follows: (total cholesterol—HDL cholesterol)/HDL cholesterol. Apolipoprotein A-1 (Apo A-1) concentration was measured using an assay kit (Nittobo, Tokyo, Japan). Lipoprotein-associated phospholipase A2 (Lp-PLA2) concentration was measured using an assay kit from R&D systems (Minneapolis, MN, USA).

### 2.6. Biochemical Analyses

The levels of plasma adiponectin, leptin, insulin, ghrelin, gastric inhibitory polypeptide (GIP), plasminogen activator inhibitor-1 (PAI-1), tumor necrosis factor-alpha (TNF-α) and monocyte chemoattractant protein 1 (MCP-1) were determined using multiplex detection kits (Bio-Rad, Hercules, CA, USA). All samples were assayed in duplicates and analyzed using the Luminex 200 LabMAP system (Luminex, Austin, TX, USA). Data analyses were carried out using the Bio-Plex Manager software version 4.1.1 (Bio-Rad, Hercules, CA, USA). Plasma glutamic oxaloacetic transaminase (GOT) and glutamic pyruvic transaminase (GPT) levels were determined using enzymatic kits (Asan Pharm. Co., Daegu, Korea). The levels of total bilirubin and gamma-glutamyltransferase (γ-GTP) were measured at the Central Laboratory (Seegene, Inc., Seoul, Korea) and analyzed using the Cobas 8000 C702 chemistry analyzer (Roche, Mannheim, Germany). The index of insulin resistance was calculated according to the homeostatic model assessment of insulin resistance (HOMA-IR) formula [23] as follows:

(fasting glucose (mmol·L^−1^) × fasting insulin (mU·L^−1^))/22.5 (1)

### 2.7. Statistical Analysis

All measured data were represented as means and standard deviations and/or standard errors. Comparisons among the placebo and d-allulose groups with respect to body composition, plasma lipid profile, T2DM-related biomarker, and indirect toxicity biomarker assessment were analyzed using analysis of covariance (ANCOVA) with independent variable as baseline and treatment. Dunnett’s two-tailed *t*-test was performed for multiple comparisons to compare the low d-allulose group or high d-allulose group with the placebo group. Significant changes between the baseline and follow-up values within groups were assessed using a paired *t*-test. A *p*-value < 0.05 was considered to be statistically significant. Data were analyzed using the SAS software (SAS institute, Inc., Cary, NC, USA).

## 3. Results

### 3.1. Study Flow

**Figure 1:**
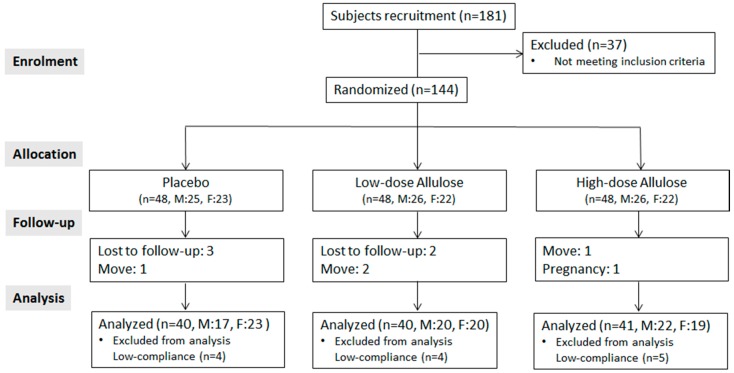
Study flow of the preliminary study for evaluating the dose-dependent effect of d-allulose. M, Males; F, Females.

Figure 1 illustrates the study flow diagram. After completing the eligibility testing for inclusion criteria for 181 candidates, 144 eligible individuals were enrolled as subjects in the present study. After 12 weeks, 10 out of the 144 subjects dropped out due to personal reasons. Further, we excluded 13 subjects because of low compliance (criterion < 80%). Compliance was determined by withdrawal rate of the test materials (placebo and d-allulose) we supplied. Thus, data from 121 subjects were finally analyzed for evaluating the efficacy of d-allulose supplementation.

### 3.2. Baseline Clinical Characteristics and Nutrient Intake

The baseline characteristics of volunteers who completed the randomized controlled trial (*n* = 121) are shown in Table 2. In both male and female subjects, there were no significant differences in age, height, systolic BP, diastolic BP, and fasting blood glucose (FBG) among all groups (Table 2). Physical activity measurement and analysis of 24-h dietary recalls from the subjects indicated no significant differences between baseline and follow-up values within each group and among the three groups (Table 3).

### 3.3. Body Composition

Table 4 shows changes in the body composition including body weight, BMI, body fat percentage (BFP), body fat mass, lean body mass, circumference of waist and hip, WHR, systolic BP, and diastolic BP. There were significant differences in BMI, BFP, and body fat mass among all the three groups after the trial. Multiple comparison tests showed that d-allulose supplementation decreased BFP and body fat mass compared to the placebo group. Moreover, high-dose d-allulose supplementation significantly lowered BFP and body fat mass as well as BMI. Comparison of the body composition-related markers before and after (follow up) revealed that body weight, BMI, BFP, and body fat mass were significantly decreased following d-allulose supplementation.

Table 5 shows the results of abdominal fat area analysis using abdominal CT scan for all three groups before and after (follow up) taking d-allulose and placebo. Comparison of total fat area exhibited no significant differences by d-allulose supplementation (ANCOVA, *p* = 0.0520), but the comparison between the high-dose d-allulose group and placebo control group revealed a significant decrease in total fat area (ANCOVA, *p* = 0.0154). However, when subcutaneous fat areas were compared, d-allulose was observed to have a significant effect (ANCOVA, *p* = 0.0103). Particularly, the high-dose d-allulose significantly decreased subcutaneous fat area compared to the placebo group (Dunnett’s two tailed *t*-test, *p* = 0.0102). The visceral fat area was not significantly altered by d-allulose supplementation.

### 3.4. Plasma Lipid Profiles

According to the 2015 Korean Guidelines for management of dyslipidemia, cut-off levels of risk in plasma lipids were as follows [24]: triglyceride ≥ 200 mg/dL (2.3 mmol/L), total-cholesterol ≥ 240 mg/dL (6.2 mmol/L), LDL-cholesterol ≥ 190 mg/dL (4.1 mmol/L). At baseline and follow-up supplementation, the levels of plasma lipids were lower than the cut-off level in all three groups (Table 6). Compared to the baseline, no significant differences in plasma lipids were observed in the three groups.

### 3.5. Blood, Plasma Glucose, and Related Biomarkers

Diabetes-associated blood indices for all groups revealed no significant differences. No significant differences were observed when the paired *t*-test was used (Table 7).

### 3.6. Plasma Adipokines and Indirect Markers of Hepatic and Renal Function

Table 8 shows the levels of plasma adipokines that are synthesized by adipocytes via regulation of energy, glucose homeostasis, and inflammation. No differences were observed among the three groups using ANCOVA and *t*-test. Plasma glutamic oxaloacetic transaminase (GOT) and glutamic pyruvic transaminase (GPT) levels, the indirect markers of hepatic function, were not observed to be significantly different among the three groups using ANCOVA and *t*-test (Table 9). Also, levels of plasma albumin and creatinine, the indirect markers of renal function, along with total bilirubin and r-GTP were not significantly different among the three groups.

## 4. Discussion

Many studies have suggested that d-allulose reduces body fat by regulating lipid metabolism in animal models [18,19]. Hayashi et al. conducted a clinical trial to examine the anti-obesity effects of d-allulose on human subjects [20]. In their study, d-allulose supplement reduced body weight, body fat percentage, and waist circumference. However, the study had some limitations. First, the sample size in their study (Males: 17, Females: 17) was too small to confirm statistical significance. Second, they used high fructose-corn syrup (HFCS) as placebo material, which can induce and/or accelerate obesity. As such, our study was designed and performed in order to address these limitations. Our results provide useful information regarding the approximate daily dosage of d-allulose for the reduction of body fat in overweight or obese subjects, although body composition measurement was performed using bioelectrical impedance analysis (BIA) instead of dual energy X-ray absorptiometry (DEXA).

The placebo control, low d-allulose, and high d-allulose groups were similar in their baseline characteristics, such as age, BMI, BFP, WHR, and FBG. There were no significant differences in the energy and nutrient intake between the three groups during the trial. Thus, d-allulose supplementation did not affect energy and nutrient intake in overweight or obese subjects.

Multiple comparison analysis showed that d-allulose supplementation in overweight or obese subjects led to a significant decrease in body fat mass, BMI, and body fat percentage. In comparing results before the study and during the follow up to the study, d-allulose supplementation was observed to reduce body weight, body fat mass, BMI, and body fat percentage. In addition, multiple comparison analysis of CT scan results indicated that the total abdominal fat area and subcutaneous fat area were significantly decreased following high allulose supplementation. In comparing results before the study and during the follow up to the study, low dose d-allulose reduced the total abdominal fat area only; however, high dose d-allulose reduced both the total abdominal fat area and subcutaneous fat area. For an accurate assessment of abdominal fat, a CT scan was used to make measurements directly at the umbilicus [25]. Although a CT scan is an expensive form of measurement and requires complicated techniques for assessing body composition, it has proven to be more accurate than anthropometry measurement or BIA [26].

In animal studies, d-allulose supplementation has been observed to improve obesity-related diseases [18,27,28]. One of these studies suggested that 5% d-allulose supplement in a high-fat diet for 16 weeks normalized the body weight and body fat mass in diet-induced obese mice [18]. Based on this study, d-allulose suppressed the dietary fat absorption by regulating the gene expression in the small intestine and increased fecal lipid contents. Also, d-allulose promoted the lipid oxidation in epididymal white adipose tissue. However, in the present study with human subjects, d-allulose supplementation for 12 weeks reduced body fat mass, body fat percentage, and subcutaneous fat (SAT) area while there was no significant reduction in visceral fat (VAT) area. These differential effects of d-allulose could be partly explained by differences in supplementation periods of d-allulose, species, and metabolic rate between SAT and VAT. In general, although both SAT and VAT were significantly correlated with metabolic risk factors, blood pressure, fasting plasma glucose, triglycerides, and high-density lipoprotein cholesterol, VAT remains more strongly associated with adverse metabolic risk profiles [29]. It can be speculated that long-term supplementation of d-allulose in human subjects could be effective for reducing VAT as well as SAT. Also, in the present study involving overweight or obese subjects, plasma lipid profiles, diabetes-associated indices, and adipokine levels were not significantly different between the baseline and follow-up study. This was probably because we used BMI as the priority cut-off marker for inclusion of subjects and the average age of these overweight or obese adult subjects was not high. Thus, their plasma lipid profiles and blood glucose concentrations were maintained at the baseline, similar to normal values.

The limitations of this trial should be emphasized. Since subjects of this study were restricted to volunteers, it was difficult to represent the general population by this small sample size. In addition, sex differences may influence plasma lipid levels due to hormonal changes during the study [30], although in our study sex differences were not shown in plasma lipids, which may be due to the relatively young ages of the participants in the study. This study was a preliminary experimental trial to assess whether d-allulose could improve obesity in a dose-dependent manner and speculate the daily d-allulose dosage for overweight or obese subjects. Also, we used CT scans in order to increase the accuracy of body composition measurements, however, they can measure only abdominal fat area. Accordingly, another main trial is underway, in which we will confirm the appropriate effective dosage and body fat reduction function of d-allulose by using DEXA, which provides whole body fat mass levels more accurately [31].

## 5. Conclusions

In conclusion, despite some limitations, we demonstrated that d-allulose is able to reduce body fat mass in overweight or obese subjects. Further, the preliminary study also indicates that the effects of d-allulose supplementation are likely to be dose-dependent.

## Figures and Tables

**Table 1:** Composition of test drinks used in the preliminary trial for dose-dependent effect of d-allulose in overweight or moderately obese human subjects.

	Placebo (Sucralose)	Low-Dose Allulose	High-Dose Allulose
	Unit: g/30mL		
Sucralose	0.012		
Allulose		4	7
Citric acid	0.024	0.024	0.051
Malic acid	0.006	0.006	0.015
Sodium citrate	0.006	0.006	0.024
Flavor	0.036	0.036	0.030
Purified water	29.922	25.928	22.880
Calorie (Kcal)	0	0	0

Placebo, Sucralose (0.012 g × 2 times/day); Low-allulose, Low-dose allulose (4 g × 2 times/day); High-allulose, High-dose allulose (7 g × 2 times/day).

**Table 2:** Baseline characteristics of the three groups of overweight or obese subjects who participated in a dose-dependent efficacy test of d-allulose.

	Placebo		Low-Allulose		High-Allulose	
	Male	Female	Male	Female	Male	Female
*n*	22	18	20	20	22	19
Age (year)	26.09 ± 4.32	25.72 ± 7.08	26.40 ± 3.75	30.40 ± 8.93	25.55 ± 4.14	27.74 ± 8.51
Height (cm)	174.95 ± 6.06	162.43 ± 4.62	174.87 ± 5.08	163.10 ± 6.30	175.17 ± 4.48	161.39 ± 4.99
Systolic BP (mmHg)	135.41 ± 13.18	118.50 ± 14.78	132.50 ± 15.10	120.05 ± 9.48	137.09 ± 11.38	114.26 ± 9.84
Diastolic BP (mmHg)	77.36 ± 10.28	66.33 ± 13.41	75.95 ± 9.95	72.45 ± 9.94	79.73 ± 10.41	67.37 ± 8.67
FBG (mg/dL)	100.24 ± 11.28	96.95 ± 11.53	100.45 ± 10.13	101.20 ± 10.80	101.64 ± 10.19	102.68 ± 10.46

Values represent the mean ± SD (standard deviation). Placebo, Sucralose (0.012 g × 2 times/day); Low-allulose, Low-dose allulose (4 g × 2 times/day); High-allulose, High-dose allulose (7 g × 2 times/day); BP, blood pressure; FBG, fasting blood glucose.

**Table 3:** Physical activity measurement and nutrient intake in the three groups of overweight or obese subjects based on 24-h dietary recall during the study.

	Placebo (M:17, F:23)		Low-Allulose (M:20, F:20)		High-Allulose (M:22, F:19)	
	Baseline	Follow Up	Baseline	Follow Up	Baseline	Follow Up
Physical activity ^1^	2.13 ± 0.10	2.18 ± 0.10	2.10 ± 0.11	2.08 ± 0.10	2.20 ± 0.11	2.17 ±0.11
Energy (kcal/day)	2440.37 ± 57.93	2450.05 ± 52.69	2433.30 ± 62.84	2438.40 ± 63.58	2421.04 ± 60.74	2429.10 ± 58.06
Protein (g/day)	112.16 ± 67.37	104.88 ± 17.66	98.96 ± 13.88	103.88 ± 17.58	104.89 ± 13.24	106.34 ± 13.59
Fat (g/day)	75.80 ± 17.07	79.81 ± 17.79	74.74 ± 16.78	77.49 ± 17.33	78.00 ± 15.78	74.79 ± 16.59
Carbohydrate (g/day)	340.05 ± 46.92	336.02 ± 44.41	340.97 ± 39.03	339.58 ± 40.86	321.87 ± 44.06	334.64 ± 45.84
Cholesterol (mg/day)	672.96 ± 196.74	734.50 ± 190.73	698.73 ± 183.52	783.43 ± 281.88	742.84 ± 175.68	734.03 ± 190.73

Values represent the mean ± SE (standard error). Placebo, Sucralose (0.012 g × 2 times/day); Low-allulose, Low-dose allulose (4 g × 2 times/day); High-allulose, High-dose allulose (7 g × 2 times/day). ^1^ Physical activity levels were scored; Low 1, Moderate 2, High 3. M, Males; F, Females.

**Table 4:** Dose-dependent effect of d-allulose supplementation for 12 weeks on BMI, BFP, body fat mass, lean body mass, WHR-related body measurements, and blood pressure in overweight or obese subjects.

	Placebo (M:17, F:23)	Low-Allulose (M:20, F:20)	High-Allulose (M:22, F:19)	*p*-Value
	Mean ± SD	Mean ± SD	Mean ± SD	
Body weight (kg)				
Baseline	77.14 ± 11.19	79.00 ± 14.72	76.63 ± 11.38	
Follow up	76.78 ± 12.20	78.02 ± 14.56 ***	75.35 ± 11.33 ^###^	0.064 ^1^
Change from baseline	−0.36 ± 1.96	−0.98 ± 1.51	−1.28 ± 1.84	
BMI (kg/m^2^)				
Baseline	26.83 ± 2.81	27.45 ± 3.21	26.79 ± 2.47	
Follow up	26.70 ± 3.14	27.13 ± 3.16 **	26.30 ± 2.49 ^###^	0.047 ^1^
Change from baseline	−0.13 ± 0.78	−0.32 ± 0.62	−0.48 ± 0.69	
Difference (Low vs. placebo) (LSmean ± SE)		−0.24 ± 0.15		0.224 ^2^
Difference (High vs. placebo) (LSmean ± SE)			−0.38 ± 0.15	0.027 ^2^
BFP (%)				
Baseline	29.61 ± 4.99	30.66 ± 4.26	29.74 ± 4.66	
Follow up	29.34 ± 4.99	29.63 ± 4.41 ***	28.73 ± 4.53 ^###^	0.027 ^1^
Change from baseline	−0.27 ± 1.16	−1.03 ± 1.44	−1.01 ± 1.51	
Difference (Low vs. placebo) (LSmean ± SE)		−0.71 ± 0.31		0.042 ^2^
Difference (High vs. placebo) (LSmean ± SE)			−0.74 ± 0.31	0.033 ^2^
Body fat mass (kg)				
Baseline	22.86 ± 5.39	24.19 ± 5.63	22.63 ± 4.20	
Follow up	22.52 ± 5.49	23.05 ± 5.36 ***	21.52 ± 4.23 ^###^	0.018 ^1^
Change from baseline	−0.34 ± 1.10	−1.14 ± 1.46	−1.11 ± 1.53	
Difference (Low vs. placebo) (LSmean ± SE)		−0.74 ± 0.31		0.033 ^2^
Difference (High vs. placebo) (LSmean ± SE)			−0.78 ± 0.30	0.021 ^2^
Lean body mass (kg)				
Baseline	54.28 ± 8.82	54.81 ± 10.82	54.01 ± 9.72	
Follow up	54.26 ± 9.67	54.97 ± 11.11	53.83 ± 9.55	0.595 ^1^
Change from baseline	−0.02 ± 1.65	0.16 ± 1.30	−0.18 ± 1.30	
Waist (cm)				
Baseline	88.40 ± 7.46	91.11 ± 9.54	89.84 ± 8.78	
Follow up	88.26 ± 7.93	90.53 ± 8.66	88.26 ± 9.01	0.298 ^1^
Change from baseline	−0.14 ± 3.20	−0.49 ± 3.61	−1.59 ± 5.74	
Hip (cm)				
Baseline	103.54 ± 6.04	103.80 ± 5.60	102.24 ± 5.52	
Follow up	102.86 ± 5.81	102.59 ± 5.35	101.65 ± 5.68	0.720 ^1^
Change from baseline	−0.68 ± 2.21	−1.21 ± 2.86	−0.60 ± 3.67	
WHR				
Baseline	0.85 ± 0.05	0.88 ± 0.07	0.88 ± 0.06	
Follow up	0.86 ± 0.06	0.88 ± 0.06	0.87 ± 0.07	0.225 ^1^
Change from baseline	0.00 ± 0.03	0.01 ± 0.04	−0.01 ± 0.06	
Systolic BP (mmHg)				
Baseline	127.80 ± 16.16	126.28 ± 13.95	126.51 ± 15.63	
Follow up	122.55 ± 12.66	122.85 ± 12.86	119.07 ± 15.38	0.194 ^1^
Change from baseline	−5.25 ± 11.42	−3.43 ± 10.31	−7.44 ± 12.55	
Diastolic BP (mmHg)				
Baseline	72.40 ± 12.89	74.20 ± 9.98	74.00 ± 11.39	
Follow up	71.20 ± 11.10	68.53 ± 10.14	68.95 ± 12.40	0.163 ^1^
Change from baseline	−1.20 ± 11.95	−5.68 ± 9.99	−5.05 ± 9.43	

Placebo, Sucralose (0.012 g × 2 times/day); Low-allulose, Low-dose allulose (4 g × 2 times/day); High-allulose, High-dose allulose (7 g × 2 times/day); LSmean, least square mean; SD, standard deviation; SE, standard error; ^1^ ANCOVA model with independent variable as baseline and treatment; ^2^ Multiple comparison tests (Dunnett’s two-tailed *t*-test); ** *p* < 0.01 and *** *p* < 0.001, paired *t*-test performed for values obtained before and after the trial in the Low-allulose group; ^###^ *p* < 0.000, paired *t*-test performed for values obtained before and after the trial in the High-allulose group; BMI, body mass index; BFP, body fat percentage; BP, blood pressure; WHR, waist-to-hip ratio; M, Males; F, Females.

**Table 5:** Dose-dependent effect of d-allulose supplementation for 12 weeks on abdominal fat area assessed using computed tomography (CT) in overweight or obese subjects.

	Placebo (*n* = 26)		Low-Allulose (*n* = 29)		High-Allulose (*n* = 26)		*p*-Value
	M: 17 (65%)	F: 9 (35%)	M: 17 (59%)	F: 12 (41%)	M: 15 (55%)	F: 12 (45%)	
	Mean ± SD		Mean ± SD		Mean ± SD		
Total abdominal fat (cm^2^)							
Baseline	322.85 ± 100.33		378.94 ± 107.39		333.83 ± 75.93		
Follow up	322.95 ± 100.59		364.64 ± 101.67 *		312.53 ± 72.35 ^###^		0.052 ^1^
Change from baseline	0.11 ± 30.61		−14.31 ± 30.89		−21.31 ± 30.45		
Difference (Low vs. placebo) (LSmean ± SE)			−9.78 ± 8.29				0.242 ^2^
Difference (High vs. placebo) (LSmean ± SE)					−20.51 ± 8.276		0.015 ^2^
Subcutaneous fat (cm^2^)							
Baseline	215.53 ± 64.78		254.15 ± 84.87		229.45 ± 72.59		
Follow up	214.87 ± 66.28		246.89 ± 79.37		208.86 ± 66.66 ^###^		0.010 ^1^
Change from baseline	−0.67 ± 21.13		−7.26 ± 26.31		−20.59 ± 24.80		
Difference (Low vs. placebo) (LSmean ± SE)			−2.76 ± 6.42				0.874 ^2^
Difference (High vs. placebo) (LSmean ± SE)					−18.54 ± 6.47		0.010 ^2^
Visceral fat (cm^2^)							
Baseline	107.31 ± 54.46		124.79 ± 40.52		104.38 ± 36.05		
Follow up	108.09 ± 58.64		117.74 ± 34.38		103.67 ± 35.30		0.446 ^1^
Change from baseline	0.77 ± 16.54		−7.05 ± 21.59		−0.72 ± 16.52		

Placebo, Sucralose (0.012 g × 2 times/day); Low-allulose, Low-dose allulose (4 g × 2 times/day); High-allulose, High-dose allulose (7 g × 2 times/day); LSmean, least square mean; SD, standard deviation; SE, standard error; ^1^ ANCOVA model with independent variable as baseline and treatment; ^2^ Multiple comparison tests (Dunnett’s two-tailed *t*-test); * *p* < 0.05, paired *t*-test performed for values obtained before and after the trial in the Low-allulose group; ^###^ *p* < 0.000, paired *t*-test performed for values obtained before and after the trial in the High-allulose group. M, Males; F, Females.

**Table 6:** Dose-dependent effect of d-allulose supplementation for 12 weeks on lipid profiles in overweight and obese subjects.

	Placebo (M:17, F:23)	Low-Allulose (M:20, F:20)	High-Allulose (M:22, F:19)	*p*-Value
	Mean ± SD	Mean ± SD	Mean ± SD	
Triglyceride (mmol/L)				
Baseline	2.00 ± 0.80	2.25 ± 1.05	2.29 ± 0.90	
Follow up	2.17 ± 1.41	2.26 ± 1.13	2.10 ± 0.82	0.160 ^1^
Change from baseline	0.17 ± 0.90	0.15 ± 0.70	−0.19 ± 0.72	
Total-C (mmol/L)				
Baseline	4.82 ± 0.68	5.14 ± 1.01	5.08 ± 1.02	
Follow up	4.82 ± 0.96	4.91 ± 1.08	5.15 ± 0.90	0.338 ^1^
Change from baseline	0.00 ± 1.07	−0.23 ± 1.01	0.07 ± 0.76	
HDL-C (mmol/L)				
Baseline	0.77 ± 0.02	0.76 ± 0.03	0.85 ± 0.02	
Follow up	0.78 ± 0.02	0.74 ± 0.02	0.86 ± 0.03	0.054 ^1^
Change from baseline	0.01 ± 0.02	−0.02 ± 0.03	0.01 ± 0.03	
Non-HDL-C (mmol/L)				
Baseline	4.05 ± 0.11	4.38 ± 0.16	4.23 ± 0.16	
Follow up	4.04 ± 0.15	4.17 ± 0.18	4.29 ± 0.15	0.561 ^1^
Change from baseline	−0.01 ± 0.17	−0.21 ± 0.17	0.06 ± 0.12	
LDL-C (mmol/L)				
Baseline	3.65 ± 0.11	3.93 ± 0.16	3.78 ± 0.16	
Follow up	3.60 ± 0.17	3.71 ± 0.18	3.87 ± 0.15	0.45 ^1^
Change from baseline	−0.05 ± 0.17	−0.21 ± 0.17	0.10 ± 0.13	
Apo A-1 (mg/dL)				
Baseline	102.67 ± 5.25	124.97 ± 1.97	137.41 ± 2.71	
Follow up	105.26 ± 7.31	119.99 ± 3.74	138.51 ± 2.70	0.180 ^1^
Change from baseline	2.58 ± 6.23	−4.97 ± 4.11	1.10 ± 3.01	
Lp-PLA2 (ng/mL)				
Baseline	5.46 ± 2.47	7.38 ± 4.07	5.53 ± 3.31	
Follow up	6.21 ± 2.78	8.19 ± 4.35	5.90 ± 3.66	0.228 ^1^
Change from baseline	0.76 ± 2.73	0.81 ± 4.52	0.37 ± 2.57	
AI				
Baseline	0.84 ± 0.04	0.85 ± 0.05	0.83 ± 0.05	
Follow up	0.80 ± 0.16	0.83 ± 0.12	0.83 ± 0.05	0.601 ^1^
Change from baseline	0.04 ± 0.25	−0.02 ± 0.12	0.00 ± 0.05	
FFA (uEq/dL)				
Baseline	442.87 ± 59.62	484.82 ± 47.12	454.72 ± 51.22	
Follow up	446.62 ± 59.25	489.12 ± 40.23	455.78 ± 55.55	0.803 ^1^
Change from baseline	3.75 ± 66.20	4.30 ± 45.78	1.06 ± 59.37	

Placebo, Sucralose (0.012 g × 2 times/day); Low-allulose, Low-dose allulose (4 g × 2 times/day); High-allulose, High-dose allulose (7 g × 2 times/day); M, Males; F, Females; SD, standard deviation; C, cholesterol; HDL, High-density lipoprotein; LDL, Low-density lipoprotein; Apo A-1, Apolipoprotein A-1; Lp-PLA2, Lipoprotein-associated phospholipase A2; AI, Atherogenic index; FFA, free fatty acids; ^1^ ANCOVA model with independent variable as baseline and treatment.

**Table 7:** Dose-dependent effect of d-allulose supplementation for 12 weeks on fasting blood glucose, HbA1c, plasma glucose, insulin, HOMA-IR, ghrelin, GIP, and PAI-1 in overweight or obese subjects.

	Placebo (M:17, F:23)	Low-Allulose (M:20, F:20)	High-Allulose (M:22, F:19)	*p*-Value
	Mean ± SD	Mean ± SD	Mean ± SD	
FBG (mg/dL)				
Baseline	97.89 ± 10.86	100.93 ± 10.19	102.12 ± 10.20	
Follow up	100.45 ± 17.76	97.40 ± 9.68	101.05 ± 17.26	0.489 ^1^
Change from baseline	2.55 ± 18.39	−3.53 ± 12.44	−1.07 ± 19.93	
HbA1c (%)				
Baseline	5.39 ± 0.29	5.49 ± 0.35	5.50 ± 0.29	
Follow up	5.36 ± 0.29	5.45 ± 0.31	5.49 ± 0.32	0.583 ^1^
Change from baseline	−0.03 ± 0.23	−0.04 ± 0.23	−0.01 ± 0.22	
Plasma glucose (mmol/L)				
Baseline	5.17 ± 0.40	5.25 ± 0.0	5.34 ± 0.47	
Follow up	5.17 ± 0.52	5.12 ± 0.63	5.21 ± 0.63	0.814 ^1^
Change from baseline	0.01 ± 0.50	−0.13 ± 1.16	−0.13 ± 0.51	
Insulin (ng/mL)				
Baseline	1.00 ± 0.60	1.17 ± 0.83	1.20 ± 1.05	
Follow up	1.09 ± 0.83	1.04 ± 0.73	1.33 ± 1.56	0.431 ^1^
Change from baseline	0.09 ± 0.73	−0.13 ± 0.83	0.13 ± 1.22	
HOMA-IR				
Baseline	2.34 ± 1.35	2.82 ± 2.16	2.78 ± 2.71	
Follow up	2.37 ± 1.68	2.43 ± 1.71	2.71 ± 2.64	0.747 ^1^
Change from baseline	0.03 ± 1.47	−0.40 ± 2.11	−0.06 ± 2.48	
Ghrelin (pg/mL)				
Baseline	199.10 ± 68.76	304.56 ± 162.86	208.04 ± 109.80	
Follow up	203.81 ± 75.29	288.31 ± 139.16	206.36 ± 94.09	0.753 ^1^
Change from baseline	4.71 ± 41.92	−16.25 ± 120.94	−1.64 ± 72.51	
GIP (pg/mL)				
Baseline	62.92 ± 54.04	113.71 ± 54.70	92.18 ± 49.97	
Follow up	77.06 ± 49.96	118.23 ± 54.84	95.47 ± 51.02	0.160 ^1^
Change from baseline	13.79 ± 46.55	2.02 ± 242.35	3.29 ± 66.19	
PAI-1 (ng/mL)				
Baseline	6.00 ± 2.94	8.55 ± 3.16	7.15 ± 3.03	
Follow up	5.27 ± 3.02	7.64 ± 4.10	6.26 ± 2.99	0.565 ^1^
Change from baseline	−0.73 ± 2.92	−0.90 ± 2.95	−0.89 ± 2.89	

Placebo, Sucralose (0.012 g × 2 times/day); Low-allulose, Low-dose allulose (4 g × 2 times/day); High-allulose, High-dose allulose (7 g × 2 times/day); M, Males; F, Females; FBG, Fasting blood glucose; HbA1c, Hemoglobin A1c; HOMA-IR, Homeostasis model assessment of insulin resistance; GIP, Gastric inhibitory polypeptide; PAI-1, Plasminogen activator inhibitor-1. TNF-α, tumor necrosis factor-alpha; ^1^ ANCOVA model with independent variable as baseline and treatment.

**Table 8:** Dose-dependent effect of supplementation of d-allulose for 12 weeks on plasma adipokine levels in overweight or obese subjects.

	Placebo (M:17, F:23)	Low-Allulose (M:20, F:20)	High-Allulose (M:22, F:19)	*p*-Value
	Mean ± SD	Mean ± SD	Mean ± SD	
Leptin (ng/mL)				
Baseline	2.79 ± 2.20	3.63 ± 2.54	2.67 ± 2.23	
Follow up	2.50 ± 2.68	3.78 ± 2.31	2.86 ± 2.46	0.231 ^1^
Change from baseline	−0.29 ± 1.48	0.15 ± 2.05	0.19 ± 1.08	
Resistin (ng/mL)				
Baseline	8.45 ± 4.44	7.09 ± 3.89	6.67 ± 2.86	
Follow up	9.28 ± 4.84	7.16 ± 3.82	7.24 ± 2.90	0.063 ^1^
Change from baseline	0.82 ± 2.11	−0.09 ± 1.15	0.57 ± 1.30	
Adiponectin (mg/mL)				
Baseline	3.46 ± 2.04	4.81 ± 3.88	3.85 ± 3.05	
Follow up	3.30 ± 1.98	4.44 ± 3.54	3.37 ± 2.48	0.558 ^1^
Change from baseline	−0.16 ± 2.14	−0.37 ± 2.33	−0.48 ± 2.03	
TNF-alpha (pg/mL)				
Baseline	13.18 ± 4.37	13.93 ± 3.36	13.16 ± 2.81	
Follow up	13.02 ± 2.71	13.98 ± 4.17	13.24 ± 2.43	0.611 ^1^
Change from baseline	−0.16 ± 3.10	0.04 ± 3.51	0.08 ± 3.27	
MCP 1 (pg/mL)				
Baseline	23.71 ± 8.69	30.50 ± 10.06	26.13 ± 8.31	
Follow up	22.23 ± 7.90	28.98 ± 11.80	26.33 ± 9.01	0.376 ^1^
Change from baseline	−1.49 ± 7.52	−1.52 ± 8.65	0.19 ± 8.58	

Placebo, Sucralose (0.012 g × 2 times/day); Low-allulose, Low-dose allulose (4 g × 2 times/day); High-allulose, High-dose allulose (7 g × 2 times/day); TNF-alpha, tumor necrosis factor-alpha; MCP-1, monocyte chemoattractant protein 1. ^1^ ANCOVA model with independent variable as baseline and treatment. M, Males; F, Females.

**Table 9:** Dose-dependent effect of d-allulose supplementation for 12 weeks on plasma GOT, GPT, albumin, and creatinine levels in overweight or obese subjects.

	Placebo (M:17, F:23)	Low-Allulose (M:20, F:20)	High-Allulose (M:22, F:19)	*p*-Value
	Mean ± SD	Mean ± SD	Mean ± SD	
GOT (AST) (Karman/mL)				
Baseline	20.04 ± 0.96	16.76 ± 1.12	18.39 ± 0.94	
Follow up	20.04 ± 1.11	16.06 ± 1.25	18.00 ± 0.97	0.504 ^1^
Change from baseline	−0.01 ± 0.42	−0.70 ± 1.06	−0.38 ± 0.66	
GPT (ALT) (Karman/mL)				
Baseline	15.47 ± 2.02	11.58 ± 1.43	11.44 ± 1.30	
Follow up	14.25 ± 1.74	10.63 ± 1.28	10.95 ± 1.14	0.786 ^1^
Change from baseline	−1.22 ± 0.71	−0.95 ± 0.86	−0.49 ± 1.05	
Albumin (g/dL)				
Baseline	4.59 ± 0.26	4.69 ± 0.29	4.68 ± 0.21	
Follow up	4.69 ± 0.29	4.76 ± 0.36	4.82 ± 0.25	0.965 ^1^
Change from baseline	0.10 ± 0.16	0.07 ± 0.27	0.14 ± 0.16	
Creatinine (mg/dL)				
Baseline	6.67 ± 1.00	6.34 ± 0.75	6.63 ± 0.55	
Follow up	6.92 ± 1.01	6.28 ± 0.60	6.70 ± 0.65	0.838 ^1^
Change from baseline	0.25 ± 0.30	−0.06 ± 0.55	0.08 ± 0.53	
Total Bilirubin (mg/dL)				
Baseline	0.49 ± 0.20	0.50 ± 0.29	0.49 ± 0.23	
Follow up	0.52 ± 0.19	0.56 ± 0.23	0.49 ± 0.22	0.385 ^1^
Change from baseline	0.03 ± 0.20	0.06 ± 0.30	0.00 ± 0.25	
γ-GTP (U/L)				
Baseline	25.51 ± 24.89	28.28 ± 21.40	27.02 ± 22.03	
Follow up	26.45 ± 22.88	21.13 ± 17.23	26.29 ± 25.24	0.249 ^1^
Change from baseline	0.95 ± 7.60	−7.15 ± 17.03	−0.73 ± 33.62	

Placebo, Sucralose (0.012 g × 2 times/day); Low-allulose, Low-dose allulose (4 g × 2 times/day); High-allulose, High-dose allulose (7 g × 2 times/day); M, Males; F, Females; GOT, Glutamic oxaloacetic transaminase; AST, Aspartate aminotransferase; GPT, Glutamic pyruvic transaminase; ALT: alanine aminotransferase; γ-GTP, Gamma-glutamyltransferase; ^1^ ANCOVA model with independent variable as baseline and treatment.
